# An Optical Sensor Network for Vegetation Phenology Monitoring and Satellite Data Calibration

**DOI:** 10.3390/s110807678

**Published:** 2011-08-04

**Authors:** Lars Eklundh, Hongxiao Jin, Per Schubert, Radoslaw Guzinski, Michal Heliasz

**Affiliations:** 1 Department of Earth and Ecosystem Sciences, Lund University, Sölvegatan 12, Lund, SE-223 62, Sweden; E-Mails: hongxiao.jin@nateko.lu.se (H.J.); per.schubert@nateko.lu.se (P.S.); michal.heliasz@nateko.lu.se (M.H.); 2 Department of Geography and Geology, University of Copenhagen, Øster Voldgade 10, Copenhagen K, DK-1350, Denmark; E-Mail: radosuav@op.pl

**Keywords:** optical sampling, Normalized Difference Vegetation Index (NDVI), remote sensing, spectral sensor, photosynthetically active radiation (PAR), phenology

## Abstract

We present a network of sites across Fennoscandia for optical sampling of vegetation properties relevant for phenology monitoring and satellite data calibration. The network currently consists of five sites, distributed along an N-S gradient through Sweden and Finland. Two sites are located in coniferous forests, one in a deciduous forest, and two on peatland. The instrumentation consists of dual-beam sensors measuring incoming and reflected red, green, NIR, and PAR fluxes at 10-min intervals, year-round. The sensors are mounted on separate masts or in flux towers in order to capture radiation reflected from within the flux footprint of current eddy covariance measurements. Our computations and model simulations demonstrate the validity of using off-nadir sampling, and we show the results from the first year of measurement. NDVI is computed and compared to that of the MODIS instrument on-board Aqua and Terra satellite platforms. PAR fluxes are partitioned into reflected and absorbed components for the ground and canopy. The measurements demonstrate that the instrumentation provides detailed information about the vegetation phenology and variations in reflectance due to snow cover variations and vegetation development. Valuable information about PAR absorption of ground and canopy is obtained that may be linked to vegetation productivity.

## 1. Introduction

**Figure 1.**
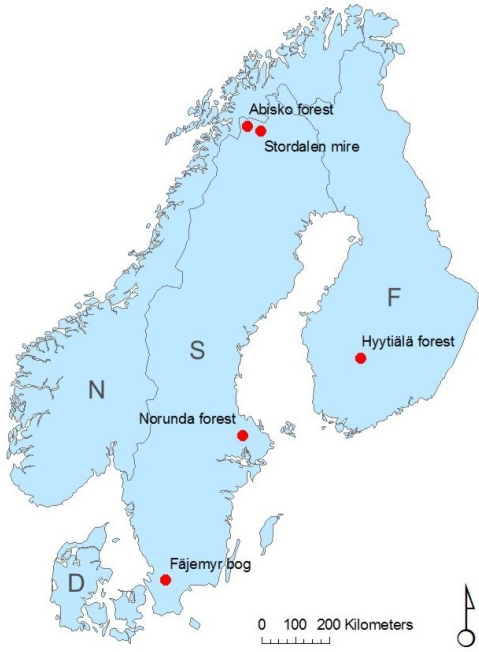
Sampling sites (red dots). D = Denmark, F = Finland, N = Norway, and S = Sweden.

Optical data sampling in broad or narrow wavelength bands provides a complement to micrometeorological measurements and vegetation sampling for estimation of biogeochemical processes. It also provides a link between measurements from Earth observation platforms and ground observations. We present an optical sampling network and data from the first year of measurement, which demonstrate the use of these data for observation of phenology and vegetation productivity (Figure 1).

Eddy covariance measurements today provide a direct way of monitoring fluxes of greenhouse gases at the ecosystem level, thereby enabling the assessment of carbon fluxes and vegetation productivity [1]. Net ecosystem exchange (NEE) is the net flux of carbon dioxide between the ecosystem and the atmosphere. The footprint of the flux measurements is a complex function of the height of measurement, the roughness of the area and vegetation, and the meteorological conditions [2]. Though the flux data have profoundly improved our ability to understand ecosystem processes, the fluxes represent the exchanges of CO_2_ between the biosphere and the atmosphere across a relatively widespread area. Data from eddy covariance towers display variations from subtle vegetation changes. However, flux tower sites are expensive to set up and are not directly comparable to satellite measurements when up-scaling flux measurement to global scale with the aid of remote sensing data. Furthermore, these complicated systems often face interruptions due to technical problems, such as power failure. Optical data and field observations can complement and aid the analysis of these fluxes, e.g., mapping the phenological development of overstorey and understorey vegetation components, and providing proxy data that can complement flux measurements during periods of missing data. Optical sampling is considerably cheaper than eddy-covariance measurements, and such sensors can hence be installed at a larger number of sites.

Normally, the size of the footprint from eddy covariance measurements approaches that of coarse-resolution satellite sensors, e.g., Terra/MODIS, with a nominal footprint area between 250 × 250 m and 1 km × 1 km, depending on wavelength band. Reflected radiation observed from these satellites can hence be directly related to eddy covariance data for upscaling purposes. Numerous studies have demonstrated the feasibility in modeling vegetation productivity, notably gross primary productivity (GPP), based on the light use efficiency concept [3–9], or statistical models [10–13]. However, satellite measurements vary due to a number of factors, including variations in illumination and viewing geometry, clouds and atmospheric conditions, variations in the pixel footprint, sensor noise, and influence of various meteorological events like snowfall, rain, and haze. Measuring with optical sensors near the ground to eliminate or minimize some of these factors will enable better understanding of the satellite measurements.

The prospect of monitoring vegetation phenology from Earth observation platforms has attracted a lot of attention. With the emergence of long time data records from Earth observation sensors it is now possible to observe variations in phenological parameters, like length of the growing season. Visible changes in vegetation phenology may act as important indicators of climatic change, as phenology responds to the effect of several physiological and biogeochemical factors of the ecosystem [14]. Current ground-based observations indicate that the recent climate-induced change in spring onset is in the order of *ca.* 2.5 days earlier per decade across Europe [15].

The difficulties in observing these subtle changes from space should not be underestimated given the normal variations in snow cover length, cloudiness, understorey vegetation, and other factors, that interfere with the remotely sensed signal during the critical periods around the beginning and end of the growing season. Nevertheless, several researchers have mapped variations in surface phenology from space data [7,16–22]. The changes observed in some of these studies appear to be larger than those observed in ground phenological datasets. Validation of phenology data can be done against a variety of reference data, including phenological data records, climate data, or physiologically modeled phenology. However, these approaches are not free from drawbacks. Phenological observations by ground observers may be of limited use since, for practical reasons, normally only a few individuals or very small areas are observed. The spatial representation of these data is therefore often uncertain, and may not be representative of the synoptic changes monitored from space [23]. Climate data generally represent larger areas, but do not represent local climatic conditions, and will not provide any information on other abiotic or biotic causes of variations in phenology. The same problems affect phenological models driven by climate data, e.g., degree day calculations, which are not complete without considering other effects [24]. Physiological processes and reasons behind the vegetation phenophase transitions are not yet fully understood, thus limiting process-based phenology modeling.

The extent to which trends observed in Earth observation data can be attributed to real changes in vegetation phenology is not fully known, and probably varies between different biomes. In sub-arctic deciduous forests, estimates of start of season based on the AVHRR and MODIS data appear to be well correlated with field observations [20], however, in Nordic coniferous forests, estimates of growing season parameters based on Terra/MODIS vegetation indices have not shown to be accurate [25]. Coniferous forests are likely more difficult to model than deciduous, and no consistent trend in seasonality was found for American coniferous forests, as opposed to nearby tundra regions [26]. Influence of snow melting, and covariation between vegetation phenology and astronomical and meteorological factors (sun elevation, snow seasonality, cloudiness *etc*.) confound the analysis, particularly for high latitude areas. Another complicating factor is that differences in data processing methodologies may generate conflicting and inconsistent results [23]. Spatially representative and consistent time series of near-ground optical measurements will enable better understanding of satellite-based phenology data, and enable improvements of processing methodology.

Phenology cameras are excellent tools for observing the greenness and leaf status of vegetation stands [27]. Data from these cameras are useful for investigating effects of weather events, and can, depending on how they are mounted, be used for observing different parts of the forest stand, e.g., for separation of overstorey and understorey variations. However, there is a lack of common standards for phenology cameras. Absolute calibration of the recorded data is seldom done, and signal and spectral drifts are problems that are difficult to quantify. Furthermore, practical problems in maintenance, mass data transfer, and image processing all limit the extensive use of phenology cameras. Cameras and radiometric sensors should be viewed as complementary tools for monitoring the phenology of vegetation stands.

Recently, networks in the support of spectral data sampling have been developed, notably SpecNet (http://specnet.info), and EUROSPEC, a recent European network established as a COST activity (http://cost-es0903.fem-environment.eu/; see a recent review by Balzarolo *et al.* [28]). While spectral measurements have been undertaken in various disciplines like biology, agriculture, and geology for decades, an important aim of these networks is to promote continuous spectral measurements in time across a variety of ecosystems. By linking spectral data with data from flux towers the networks aim at improving ecosystem monitoring, and improve understanding and upscaling efforts using satellite earth observation [29]. Sampling can be carried out in a variety of spectral, spatial and temporal resolutions, depending on the aim of the analysis. Hyperspectral sampling, with hundreds of spectral channels across the reflected optical spectrum, is useful for investigating detailed biogeochemical mechanisms, e.g., the photosynthetic process, or for formulating optimal vegetation indices. Multispectral or panchromatic measurements cannot achieve these aims but may be sufficient for validating satellite-sensed measurements of phenology or vegetation productivity. Measurements of photosynthetically active radiation (PAR) can be useful for determining vegetation phenology, and for estimating GPP (for a review, see Hilker *et al.* [30]).

The aim of the paper is to demonstrate a spectral sampling design, tested at five flux tower sites, consisting of various sensors that are relatively inexpensive, easy to maintain, and that can provide continuous year-round data. We present the theory of measurements to show that reflectance measured from our viewing geometry with large field-of-view (FOV) is close to that from infinitesimal FOV. We also show the computation of different PAR fractions, considering multiple reflections between canopy and ground. We present data from the first year of measurement (2010) to demonstrate the feasibility of the measurements for vegetation phenology and calibration, and discuss some advantages and possible errors with the type of data collection. Our analysis is carried out in relation to our efforts in calibrating satellite observations to be used for vegetation productivity and phenology. The network reported in this article represents a contribution to existing global and regional flux and spectral networks.

## 2. Infrastructure of the Spectral Network

**Figure 2.**
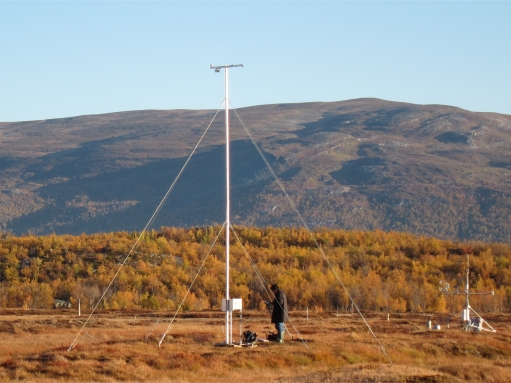
Photograph of the Stordalen mire site on September 18, 2010, showing the spectral mast (centre) and an eddy covariance flux tower (right). The oblique sensor views the ground tundra cotton-grass with a 55° off-nadir angle. The flux tower is measuring the carbon flux, and is operated by N. Roulet, McGill University, Montreal, Canada.

The network consists of five sites, located in Sweden and Finland, which range latitudes from 56°N through 69°N (Figure 1). Their location was determined by the access to existing flux towers. Optical signals were sampled continuously throughout 2010, except for some short periods of power failure. Table 1 provides details about the measurement sites. At two sites (the coniferous forests) we utilize existing high flux towers for our sensors, and at the remaining sites we have erected separate masts for the measurements (for an example, see Figure 2).

### 2.1. Multi-Spectral Sensors

We used multi-spectral sensors (SKR-1800 and SKR-1850A) from Skye Instruments Ltd, UK (http://www.skyeinstruments.com/) with two or four wavelength bands for narrow-band spectral sampling. Sensor characteristics are specified in Table 2. These sensors have photo-detectors made from gallium phosphide (GaP), gallium arsenide phosphide (GaAsP), or silicon, depending on wavelength.

The sensors measured two green bands for computation of photochemical reflectance index (PRI), and red and near-IR bands for computation of normalized difference vegetation index (NDVI), with somewhat different wavelength specifications depending on the manufacturer’s calibration. The SKR-series light sensors measure with a 25° FOV as a standard, and an additional cosine correction diffusor was provided to enable hemispherical irradiance measurements. To achieve a wider FOV than 25° for the downward-looking sensors, custom-made 60° FOV collars were mounted on top of the cosine diffusors. However, this construction attenuated the signals, and at one site (Norunda forest) we had to use the standard 25° FOV to improve the signal-to-noise (S/N) ratio.

Radiation sensors for reflectance measurement used in meteorology are generally mounted for viewing vertically towards the ground. For vegetation monitoring this may not be optimal for the following reasons: (1) the sensor may view the tower and the installations next to it; (2) the footprint size is generally small (for narrow-angle sensors/low tower); (3) the instrument may view an disproportionally large gap area between the trees; and (4) the ground right below the tower is sometimes disturbed by frequent access by observers. These problems can be avoided by directing the instrument off nadir, though these measurement results will differ from nadir observations due to anisotropic effects. However, several studies have demonstrated that off-nadir viewing might be advantageous for vegetation monitoring. For example, in agricultural crops, Demetriades-Shah and Court [31] and Aparicio *et al.* [32] demonstrated improved estimates of chlorophyll and other agronomic traits, respectively, by measuring with field instruments in off-nadir directions rather than in nadir. Also Dunham and Price [33] found differences, but only when looking in the direct backscatter region. For other azimuths they did not see any differences when estimating fraction of vegetation cover and height of grass. Gemmel and McDonald [34] investigated angular effects on estimation of canopy variables in forests using simulated and measured reflectance data. They found that discrimination of cover and leaf area index (LAI) was improved in off-nadir direction in comparison to nadir viewing. Going up in scale, Xavier and Galvão [35] showed that discrimination and mapping of Amazonian land cover types were improved in off-nadir viewing, using data from MISR satellite sensor.

We mounted downward-looking sensors at an oblique angle of 55° off nadir (Figure 2). The measurements were directed towards the dominant area in the eddy covariance flux footprint (according to the most frequent wind direction). We aimed for roughly westward or eastward azimuths to avoid either back scattering or forward scattering effects in the solar principal plane roughly along the N-S direction at noon, and to approximate the sensor view azimuth angles of most polar-orbiting satellites. Small adjustments had to be made in order to mount the mast at an easily accessible spot, moreover, with solid foundation. The upward-looking multispectral sensors measured with a cosine correction diffuser, thus sampling irradiation from the whole upper hemisphere with equal response across the sensor aperture from any direction. The downward-looking multispectral sensors measure conically, thus enabling computation of hemispherical-conical reflectance factors (HCRF, following the nomenclature by Nicodemus *et al.* [36] and Schaepman-Strub *et al.* [37]. We further note that the term “reflectance” in this paper refers to “HCRF”). Since there was a tendency for water films covering sensor aperture holes, we covered the downward-looking sensors with a small roof to protect them from rain. The geometrical properties of the downward-looking multispectral sensors (not PAR, since sensors for PAR reflection measurement were always facing vertically downward with hemispherical FOV) are given in Table 3.

Optical sensor footprint sizes vary between *ca.* 1,400 and 6,900 m^2^ for the sites with conical measurements of reflected radiation, depending on sensor FOV and height of measurements. Using a fixed sensor to completely match a flux tower footprint is impossible given that fluxes are dynamic and the footprint area is very difficult to predict [2]. We strived at placing our optical towers carefully within the target ecosystems of the flux towers in order to facility the comparison of the two measurements. The sites are homogeneous, and phenological transitions viewed by our towers occur more or less simultaneously across each flux tower footprint area.

### 2.2. PAR Sensors

Our installations also include data from broadband PAR sensors, measuring across the full visible wavelength band (400–700 nm). These measurements are often standard at flux towers for estimation of vegetation properties. To enable separation of absorbed PAR from ground vegetation and forest canopy it is necessary to measure four PAR fluxes: (1) incoming above the canopy measured by an upward-looking sensor (*PAR*_0_); (2) total reflected PAR, including the forest canopy direct reflection, ground direct reflection, and canopy transmission of the ground reflection, measured by a downward-looking sensor (*RPAR_eco_*); (3) PAR transmission through the canopy measured by an upward-looking sensor below canopy and one meter from the ground (*TPAR*); and (4) reflection from the ground measured at the same location as TPAR by a downward-looking sensor (*RPAR_g_*). Complete sets of PAR sensors have not yet been mounted at all our sites and only one site currently enables measurement of all these fluxes (Table 4). For the open sites without tree canopies (Stordalen mire and Fäjemyr bog), *RPAR_eco_* cannot be distinguished from *RPAR_g_* since it is impractical to position sensors below the canopies of herbs and mosses.

## 3. Measurement Theory

### 3.1. Reflectance and NDVI

**Figure 3.**
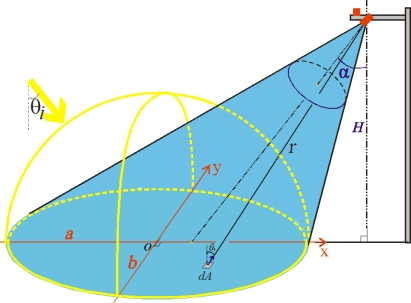
Geometry diagram of the oblique-looking sensor. The downward-looking sensor, mounted at height *H* with an oblique off-nadir angle α or around 55°, has an ellipse footprint with semi-major axis *a*, and semi-minor axis *b*. A reflection beam, with an angle *θ_r_* to the normal of the small area *dA*, reaches the sensor at a distance *r*. The yellow arrow shows the direct incoming light, which is added to the diffuse light from the upper hemisphere.

The geometry of the downward-looking reflectance sensors is shown in Figure 3. The average footprint reflectance [44] was computed by (see Appendix 1 for deductions):

(1) $$\bar{\rho }=\frac{\pi {\bar{L}}_{r}}{E}=\frac{\int_{\Omega_{A}}\pi f\left(\theta_{r}\right)\cdot d\omega^{\prime }}{\int_{\Omega_{A}}d\omega^{\prime }}=\frac{\int_{A}\pi f\left(\theta_{r}\right)\cdot \frac{\text{cos}\;(\theta_{r})}{r^{2}}\mathrm{dxdy}}{\int_{A}\frac{\text{cos}\;(\theta_{r})}{r^{2}}\mathrm{dxdy}},$$

where *L̄_r_* is the mean radiance of the area (sensor footprint) *A*, *E* is the hemispherical irradiance, *πf*(*θ_r_*) is the hemispherical-directional reflectance factor (HDRF), *dω*′ is the projected solid angle subtended by the differential area *dA* (*i.e.*, *dxdy*), and ω*_A_* is the integration range of the projected solid angle subtended by the footprint. Thus, the measured average footprint hemispherical-conical reflectance is the average of *πf*(*θ_r_*) over the projected solid angle subtended by footprint *A. πf*(*θ_r_*) is written as [36]:

(2) $$\pi f\left(\theta_{r}\right)=\int_{2\pi }d\phi \int_{0}^{\frac{\pi }{2}}f\left(\theta_{i},\theta_{r}\right)\cdot \text{sin}\left(\theta_{i}\right)\cdot \text{cos}\left(\theta_{i}\right)\cdot d\theta_{i},$$

where *f*(*θ_i_*, *θ_r_*) is the bidirectional reflectance distribution function (BRDF) at the differential area *dA* with the incoming light angle *θ_i_* and reflected light angle *θ_r_*. The spatial contribution from a Lambertian surface to the total footprint reflection decreases away from nadir towards the far end of the footprint according to a Cauchy distribution. It shows that the radiance captured by our sensors is dominated by flux originating from the closest half of the footprint area.

NDVI [38] is computed as:

(3) $$\mathrm{NDVI}=\frac{{\bar{\rho }}_{\mathrm{NIR}}-{\bar{\rho }}_{\mathrm{red}}}{{\bar{\rho }}_{\mathrm{NIR}}+{\bar{\rho }}_{\mathrm{red}}},$$

where *ρ̄_NIR_* and *ρ̄_red_* are average footprint HCRF’s in NIR and red bands, respectively.

#### 

##### Modelling Anisotropic Effects on the Footprint HCRF

**Figure 4.**
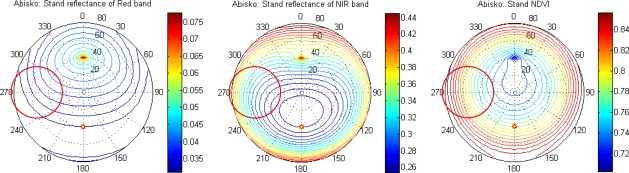
HCRF and sensor footprint at the Abisko forest stand as modeled with FRT [39]. The sensor is looking at 261° direction with an off-nadir angle of 55° and an FOV of 60°. The sun zenith angle is 40° at 180° relative azimuthal direction, shown as an asterisk. The sensor footprint ellipse is projected as a circle in the polar coordinate system (red circle).

In order to investigate the feasibility of sampling anisotropic reflectance using a wide-angle sensor we used the Kuusk and Nilson forest reflectance and transmittance (FRT) model [39] to simulate reflectance measured by our tower sensors under hemispherical-conical geometry. The model was driven with field measurements on average stand characteristics (Table 5), and ground vegetation properties. In the model, PROSPECT [40] is used for leaf optics, and 6S [41] is used for atmosphere correction, simulating both direct and diffuse sun light. The sun was fixed at a zenith angle of 40°, and simulations were made over all azimuth angles, and zenith angles varying between 0 and 80°. Figure 4 displays simulated HDRF for red and NIR bands, and for NDVI. The HDRF properties of the ground vegetation can also be given by the FRT model. The modeling results were further integrated over the footprint area to simulate HCRF, reflectance and NDVI, as measured by our tower sensors under hemispherical-conical geometry. These computations were made for one forest canopy simulation and one ground vegetation simulation separately.

**Figure 5.**
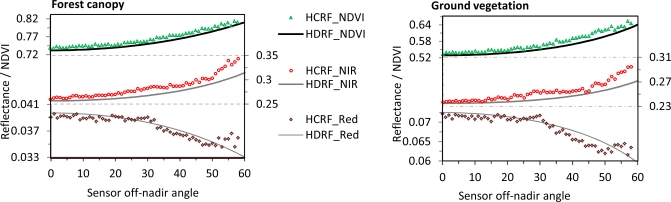
Variation of red and NIR mean reflectance (HCRF) and NDVI with sensor off-nadir angle, in comparison with HDRF values from the FRT model along 270° azimuth. The left graph shows the Abisko forest canopy reflectance and the right graph shows the reflectance from the ground vegetation of the same stand. The HCRF results are computed by using Monte-Carlo integration and the FRT model.

The simulated HDRF was integrated with a Monte-Carlo method. Since it can be shown that the FOV-subtending footprint points have a Cauchy distribution [42] given a uniform distribution of the viewing angle, *θ_r_*, Cauchy random variables were sampled to fulfill the Monte-Carlo integration (see Appendix 2 for details). Results of the modeling are given for one site (the Abisko forest) in Figure 5, with separate figures for forest canopy and ground vegetation.

Figure 5 shows that the HCRF and HDRF curves are very close to each other, indicating that almost identical results can be achieved using large FOV observations as when observing with infinitesimal FOV. This is equally true for forest canopy and ground vegetation. Due to the BRDF effects, off-nadir observations in NIR and NDVI are larger than those of nadir viewing, whereas the red band displays opposite variations. We note that there is bias in HCRF results in Figure 5 for sensor oblique angles greater than 50°. This is due to the facts that we used a 60° FOV sensor and that the FRT model does not give accurate results for viewing angles as high as 80° and above [43] (80° being the far edge of our measurements considering the off-nadir angle and the FOV; 50° + 60°/2 in our observation geometry). Because of the fact that points with over 80° viewing angle are rare in our sampling, these points contribute minimally to the integration result, and they were neglected.

### 3.2. PAR Quantities

The fractions of PAR absorbed by the forest canopy (*fAPAR_c_*), the ground (*fAPAR_g_*), and the whole ecosystem (*fAPAR_e_*), as well as PAR intercepted by the forest canopy (*fIPAR*), can be calculated from the relevant PAR components, as shown in Table 6. It should be pointed out that *fAPAR_c_* is the fraction of PAR absorbed by the forest canopy, and it is different from “green” *fAPAR_c_* absorbed by photosynthesizing leaves which can be derived from vegetation indices by statistical methods [44]. We observed high *fAPAR_c_* during the winter, and this portion of PAR, partly attributed to the long geometrical pathway due to large sun zenith angles in winter, is obviously not used for photosynthesis.

**Figure A2.**
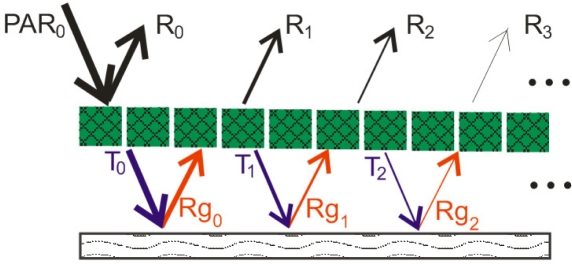
Simplified canopy model with multiple reflection and transmission of PAR. R_0_ denotes direct reflection to the above-canopy sensor, R_1_ denotes 1-time interaction with ground and then transmitted through the canopy to the sensor, and so on. T_0_ denotes direct transmitted PAR to the upward-looking sensor below the canopy, T_1_ denotes that T_0_ is reflected by the ground one time, then by the bottom of the canopy one time, and then received by the same sensor, and so on. Rg_0_, Rg_1_,…, denote the first, second,…, ground reflections.

The measured canopy PAR transmission, TPAR, contains contributions from multiple reflections between ground and canopy bottom (see Figure A2 in Appendix 3), therefore the measured transmittance *τPAR_m_* is not the “true” transmittance of the canopy. It can be shown that the true transmittance is (see Appendix 3 for details):

(4) $$\tau \mathrm{PAR}=\tau {\mathrm{PAR}}_{m}\cdot \left(1-\mathrm{rPA}R_{c}\cdot \mathrm{rPA}R_{g}\right),$$

where the pure canopy reflectance *rPAR_c_* is computed by:

(5) $$\mathrm{rPA}R_{c}=\frac{\mathrm{rPA}R_{\mathrm{eco}}-\tau \mathrm{PA}R_{m}^{2}\cdot \mathrm{rPA}R_{g}}{1-\tau \mathrm{PA}R_{m}^{2}\cdot \mathrm{rPA}R_{g}^{2}}.$$

Considering the incoming light from ground reflection that may undergo interception by canopy, we express the fraction of canopy totally-intercepted PAR as:

(6) $$\mathrm{fIPA}R=\left(1-\tau \mathrm{PAR}\right)\cdot \left(1+\tau {\mathrm{PAR}}_{m}\cdot \mathrm{rPA}R_{g}\right).$$

## 4. Data Processing

### 4.1. NDVI

Two types of calibrations were done for the narrow-band sensors by the manufacturer before delivery; absolute calibration for the hemispherical sensors and those with 60° FOV, and relative calibration for the sensors with 25° FOV. For sensors with absolute calibration, narrow band reflectance was calculated by dividing the reflected radiance *L*[μmol·s^−1^·m^−2^·sr^−1^] with the irradiance *E*[μmol·s^−1^·m^−2^], and then multiplied by *π* [sr]:

(7) $$\rho =\frac{\pi L}{E}.$$

After the reflectance was computed, NDVI was computed using Equation (3). For the sensors without absolute calibration, narrow band reflectance could not be calculated. While ratio-based vegetation indices can still be obtained from the relative sensitivity, e.g., NDVI and simple ratio, non-ratio-based vegetation index, e.g., EVI2 [48] cannot be calculated.

The data loggers recorded flux signals and internal temperature with 10-minute intervals resulting in 144 diurnal records for each channel. Daily solar noon NDVI values were calculated from these records. Temperature varies from around −20 °C to 25 °C at our test sites, and the temperature drift of the light sensors was compensated by analyzing the mid-night measurements in relation with the simultaneous temperature. This compensation is especially critical when the daytime incoming light is weak. For the Abisko forest, the incoming light at winter noon is 0.4% of summer peak values in our data, which leads to 15 times lower S/N ratio in winter than in summer based on shot noise theory [49].

### 4.2. PAR

We estimated PAR components of canopy and ground with the aim of separating tree phenology from ground vegetation phenology. For the Abisko forest site, with measurements of four PAR components, this was straight forward, using formulae in Table 6 and Equation (5). For the Norunda forest, only three PAR flux components were available, and we assumed that PAR reflectance of the forest floor was fixed. We therefore used *rPAR_g_* from transect measurements under a tree canopy in a southern Swedish forest, using a value of *rPAR_g_* of 0.059 ± 0.022 for snow-free ground. It was then possible to use Equation (5) to estimate canopy reflectance. For the remaining sites no separation of PAR reflectance was possible, and we only computed total PAR reflectance and whole ecosystem PAR absorption by using two-component measurements above the canopy.

## 5. Results

We demonstrate seasonal trajectories of NDVI, PAR reflectance and transmittance, and fractions of PAR absorption and interception. We also provide some examples of how our measurements can be used in the interpretation of vegetation phenology. Although PRI is computed for three of our sites we do not show it in this paper.

### 5.1. NDVI

#### 5.1.1. Seasonal Courses

**Figure 6.**
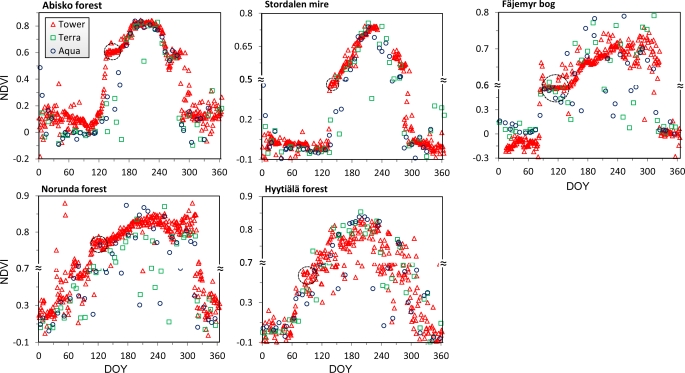
NDVI courses of five sites in 2010. Tower-based NDVI are calculated from measurements at solar noon. Terra and Aqua MODIS-derived NDVI were computed from 8-day composite reflectance. The scales are varied along the y-axes, in order to amplify subtle NDVI variations during the growing seasons. The tower-based NDVI curves expose a “shoulder” at the start of the growing season (dotted circle).

The seasonal NDVI courses of the five sites are presented in Figure 6, calculated from red and NIR measurements at solar noon, and corrected for the dark current drift bias inferred from midnight measurements. NDVI from 8-day composite MODIS surface reflectance from the Terra and Aqua platforms (original MOD09A1 and MYD09A1 data for the tower site pixels) is plotted for comparison. The seasonal variations in NDVI are large for each of our sites due to alternation of snow cover and vegetation cover. The effects of snow during the spring and autumn generally hide the subtle variations in NDVI due to vegetation phenology, such as onset and end of the season. In order to highlight these transition stages we have plotted data with varying y-axis scale to accentuate the subtle changes during the growing season (*ca.* DOY 120–280). This was particularly necessary for the evergreen coniferous forests of Norunda and Hyytiälä, and also for sites with semi-evergreen vegetation like the Fäjemyr bog and Stordalen mire.

All the curves in Figure 6 expose a short pause or “shoulder” after the abrupt increase of NDVI due to ending of the snow season, just before the beginning of the growing season. This short “shoulder” lasted for 39 days at the Fäjemyr bog (DOY 95–134), 26 days at the Norunda forest (DOY 106–132), 25 days at Abisko forest (DOY 135–160), and 6 days at the Stordalen mire (DOY 136–142). Moreover, the shoulder at the Fäjemyr bog appeared to be decreasing slightly, from 0.59 on DOY 95 to 0.57 on DOY 134. The shoulder value of at the Hyytiälä forest dropped even more, from 0.61 on DOY 83 to 0.39 on DOY 101. The shoulder period appears to be a special characteristic of the onset of the growing season in high-latitude regions, as observed at our sites.

We observed large increases in NDVI during the spring period of the five sites (Abisko forest DOY 103–135, Stordalen mire DOY 128–136, Fäjemyr bog DOY 80–95, Norunda forest DOY 100–106, and Hyytiälä DOY 58–83). By combining information from phenology camera images and weather station records we know that these are the snow melting periods.

It can be seen that satellite-based and ground-based measurements agree with each other very well (R^2^ = 0.68∼0.78, sample size N = 51∼70). However, MODIS data tend to display larger scatter during the growing season, probably due to cloud contamination that may affect satellite measurements more than the tower measurements. Tower NDVI from the Hyytiälä forest display a relatively larger scatter than the other sites, for unknown reasons. The “shoulder” is less visible in MODIS data due to the coarser temporal resolution.

#### 5.1.2. Daily variations

**Figure 7.**
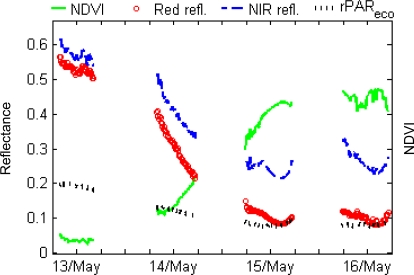
Daily trajectories in reflectance, PAR, and NDVI at Stordalen mire from May 13 (DOY 133) to May 16 (DOY 136). Daily NDVI increased by 0.2 on May 14 and May 15. The reflectance in red, NIR and PAR decreased simultaneously with the largest decrease occurring for the red reflectance. The labels are shown at the 12:00 location of the x-axis, and the tick interval is 6 hours.

To further analyze the striking increase in NDVI before the shoulder period, probably caused by the melting of snow (Figure 6), we generated plots of daily reflectance and NDVI. Figure 7 gives an example from the Stordalen mire. It shows data from four days in May 2010, when the land surface NDVI increased from 0.04 on May 13 to 0.45 on May 16. Daytime NDVI changed by almost 0.2 at 7:50 to 16:20 during the 14th and 15th. The corresponding sun zenith angle variations between 48° and 62° may contribute to NDVI variations of less than 0.02, according to FRT model simulations.

### 5.2. PAR

#### 5.2.1. Reflectance and Transmittance

**Figure 8.**
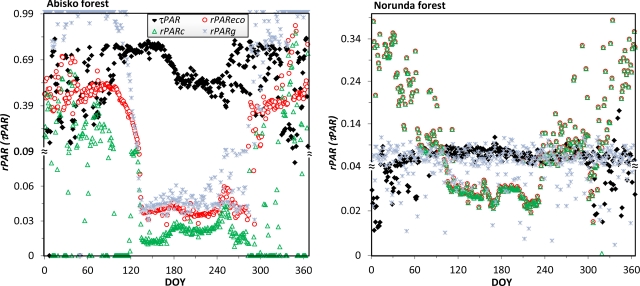
The annual courses of canopy PAR transmittance, ecosystem PAR reflectance, canopy PAR reflectance, and ground PAR reflectance at the Abisko (left) and Norunda (right) forests. Note the varied scale for the y-axis which is used to amplify the reflectance and transmittance changes during the growing season and transition periods snow-to-vegetation and vegetation-to-snow.

We demonstrate the effect of decomposing ecosystem PAR reflection into flux reflected from pure canopy and flux from the ground at two forest sites: Abisko birch forest and Norunda coniferous forest (Figure 8). We also show the “true” PAR transmittance, *τPAR*. This quantity is smaller than the measured *τPAR_m_*, which contains a portions of the ground reflectance. The difference between *τPAR* and *τPAR_m_* was substantial during the snow season for the Abisko forest, but there was no obvious difference for the Norunda forest. We will not show *τPAR_m_* results here.

PAR transmittance at Abisko forest showed a clear seasonal pattern, with the lowest values appearing in the middle of the growing season (DOY 183–242). At Norunda forest, the seasonality of PAR transmittance was considerably weaker, with no clear minimum. PAR reflectance was lowest during DOY 135–249 at the Abisko forest, and during DOY 110–229 at the Norunda forest. In the Abisko forest, the subtle but regular variations in PAR reflectance during these periods largely corresponded to the fluctuation in PAR transmittance (Figure 8, left).

In the Norunda forest, strong seasonal patterns were only observed from PAR reflectance of the whole ecosystem and of the canopy, and these two quantities closely followed each other (Figure 8, right). The period of high *vs.* low reflectance at Norunda forest clearly indicated the snow period (before DOY 64, and after DOY 315), and the snow-free period (DOY 110–229). During the snow period, the reflectance of the canopy was persistently higher (over 0.2), but the variability of the two reflectances was quite large due to the coexistence of green canopy and snow on the branches.

In the Abisko forest, the snow season can be easily identified from the ground PAR reflectance, *rPAR_g_*, which approximates to one for the snow period (before DOY 87 and after DOY 293), and falls below 0.05 during the snow-free period (DOY 135–280). As a contrast, the pure canopy reflectance, *rPAR_c_*, should always hover around a low value, since both green leaves in summer and bare branches in winter have low reflectance. However, we observed relative higher *rPAR_c_* for some intermittent periods in winter. These high reflectances were most likely caused by short periods of snow on the branches.

The seasonal trajectory of the fraction of transmitted PAR, *τPAR*, reflects foliage conditions of the tree canopy. In the deciduous Abisko forest, *τPAR* fluctuates regularly, with low values during the full-foliage stage (DOY 183–242), and high values during the bare-branch stage (before DOY 183 and after DOY 280). This pattern of fluctuation is less obvious at the Norunda forest, since the canopy there is evergreen. At this site, *τPAR* variations are clearly more influenced by snow on the branches than by plant phenology variations.

Turning points in all of the PAR curves at Abisko forest nearly match those of the NDVI curves in Figure 6, e.g., at DOY 135, 160, 183, 242, and 280. At Norunda forest, only ecosystem and canopy reflectance data match those in the NDVI curve, e.g., at DOY 64, 106, 198, 235, and 305. Two of these correspond to observations from phenology camera images: canopy snow cover disappeared at DOY 64, and the ground snow cover disappeared at DOY 106.

#### 5.2.2. fIPAR and fAPAR

**Figure 9.**
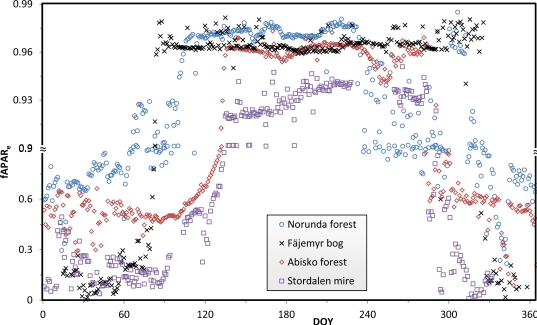
Fraction of ecosystem absorbed PAR at four tower sites. Note that *fAPAR_e_* below and above 0.9 are shown in different scales in order to emphasize variations during the growing season.

The fraction of PAR absorbed by the whole ecosystem, *fAPAR_e_,* was calculated for four of the sites, and is shown in Figure 9. The curves display the variation in seasonal length across the different sites. As expected, the absorbed PAR is higher during the snow-free season and lower during the snow season.

The dates of end and onset of the snow season and the length of the growing seasons vary across the sites, as a consequence of differences in latitude and vegetation type. However, the magnitudes of *fAPAR_e_* during the snow-free period are very similar (*ca.* 0.93–0.98) at all the sites. Again, we have used a variable y-axis scale to highlight the subtle changes of *fAPAR_e_* during this period.

**Figure 10.**
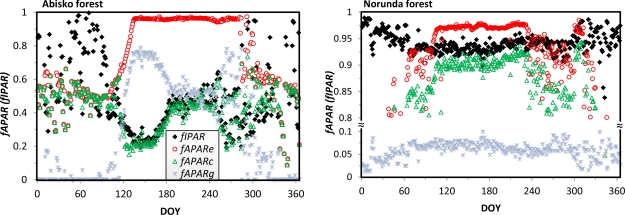
fIPAR and fAPAR at the Abisko (left) and Norunda (right) forests.

The intercepted PAR, fIPAR, was computed from Equation (6), and the three fAPAR quantities were computed according to Table 6. These four quantities are illustrated for the Abisko and Norunda forests in Figure 10. The fIPAR was slightly greater than the canopy-absorbed PAR within the snow-free period for the two forests. The total absorbed PAR was very stable throughout the snow-free season, and the two components, *fAPAR_c_* and *fAPAR_g_*, varied in opposite directions during this period. This is easily seen for the Abisko forest, where the turning point on the *fAPAR_e_* curve is very sharp on DOY 135. From this date until DOY 160, *fAPAR_c_* and *fAPAR_g_* hovered at ∼0.22 and ∼0.74 respectively. After DOY 160, *fAPAR_g_* decreased and *fAPAR_c_* increased at the same rates, until DOY 183. Then *fAPAR_g_* leveled out at ∼0.49, and *fAPAR_c_* leveled out at ∼0.46 until DOY 242. After that date their directions diverged, but with considerable noise. No obvious trends in *fAPAR_c_* and *fAPAR_g_* were observed after DOY 280 until the end of the year, neither in the period from the start of the year to DOY 87.

Periods with different fraction quantities indicate different phenological stages of the forest canopy and ground vegetation, and differences in PAR absorption reflect different productivities. Variations in *fAPAR_e_* indicate the seasonality status of the whole ecosystem, and the signal is composed of both variations in vegetation cover (high *fAPAR_e_*) and snow cover (low *fAPAR_e_*). In the growing season, increases in the absorbed PAR may imply increasing productivity, e.g., for the canopy. However, for the ground vegetation, the decrease in *fAPAR_g_* does not necessarily mean that the ground vegetation productivity is decreasing. For example, during the period of DOY 160–183 in the Abisko forest, the decrease is caused by an increase in canopy-intercepted PAR.

The seasonal pattern of land surface phenology (*i.e.*, snow and vegetation variations) in the Norunda forest can be identified from the course of total ecosystem-absorbed PAR, and a similar seasonal pattern is also seen from the pure canopy-absorbed PAR. However, these variations do not reflect the phenology of the coniferous trees. Neither is it possible to differentiate between the phenological stages of the coniferous trees and of the ground vegetation at the Norunda forest based on our measurements (Figure 10, right), since the lack of measurements of ground reflectance precludes determination of the annual course of ground absorbed PAR.

### 5.3. Correlation between PAR and NDVI

**Figure 11.**
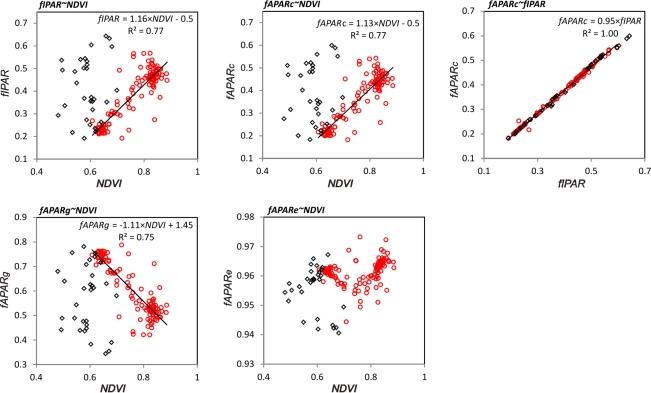
Relationships between fractions of PAR and NDVI in the Abisko forest during the snow free season (DOY 135–280). Red circles denote measurements for DOY 135–249, black diamonds for DOY 250–280.

There was a linear relationship (R^2^ = 0.77, N = 133) between the fraction of canopy-absorbed PAR, *fAPAR_c_* and NDVI, for the Abisko forest from the start of the growing season until before the leaf discoloration period (DOY 135–249) (Figure 11). The linear relationship deteriorated during the leaf discoloration and defoliation period (DOY 250–280), when NDVI values were generally decreasing. Similar results are also obtained for the relationship between the canopy-intercepted PAR, *fIPAR*, and NDVI. *fAPAR_c_* was consistently 5% lower than *fIPAR* throughout the snow free period (*fAPAR_c_* = 0.95· *fIPAR*; R^2^ = 1.00, N = 133). The fraction of ground absorbed PAR, *fAPAR_g_*, showed a negative relationship with NDVI, which reflects that the ground vegetation absorbed less and less PAR during the course of the NDVI increase, since an increasing amount of PAR was absorbed by the forest canopy before reaching the ground. This PAR competition lasted until the tree leaf discoloration and defoliation, evidenced by the NDVI increase during DOY 259–282 (Figure 6, Abisko forest). This short term recovery is attributed to the ground vegetation. There was no linear relationship between the total PAR absorbed by the ecosystem, *fAPAR_e_*, and NDVI. The variability of *fAPAR_e_* during the snow free period (standard deviation, std = 0.004) is much smaller than the variability of other PAR fractions (std = 0.10 − 0.11).

### 5.4. Relationship with Phenology and Environmental Data

**Figure 12.**
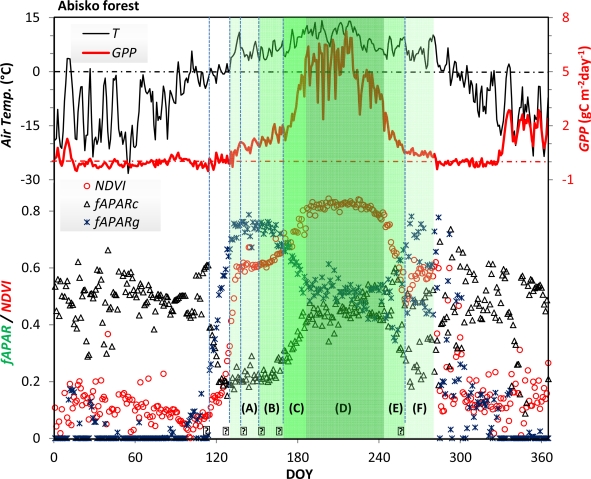
Comparison of NDVI, *fAPAR_c_*, and *fAPAR_g_* with diurnal GPP and air temperature. Dashed vertical lines show the ground-observed phenology events (Filled circles 1–6): 1. DOY 114 (April 24) half snow, half bare ground, 2. DOY 130 (May 10) end of snow melting, 3. DOY 139 (May 19) birch winter-bud to bud swelling, 4. DOY 148 (May 28) bud swelling to bud growth begins, 5. DOY 168 (June 17) full leaf development, 6. DOY 259 (September 16) all the leaves have become yellowish. Green fields denote vegetation periods (labeled A–F); see the text for explanation.

In addition to the optical measurements at the Abisko forest, we have analyzed other observations of vegetation phenology and productivity, including eddy covariance measurements, air temperature records, digital camera photographs, and manual observations by observers at the Abisko Scientific Research Station (*ca.* 1.3 km away from our site). These data are shown in Figure 12. GPP was estimated at this site from net ecosystem exchange (NEE) flux measured by an open-path infrared CO_2_ analyzer (Li-Cor 7500, Li-Cor Inc., USA) and a three-dimensional sonic anemometer (Gill R3, Gill Instruments, UK). The partition algorithm is described by Reichstein *et al*. [50]. Air temperature was measured at the height of 2 meters using a Vaisala WXT 510 Weather Transmitter (Vaisala Oyj, Finland).

The site is complex in the sense that it consists of sparse deciduous canopy on top of a partly evergreen understorey layer. As explained in the previous sections, the seasonality of snow, understorey, and overstorey are confounded, creating a complex annual reflectance curve. GPP starts to increase above zero at almost the same date in spring as the temperature does, and it decreases to zero in the autumn, slightly before the temperature. The timings of phenophase transitions from ground observations and phenology camera images are drawn as vertical lines (labeled 1–6 in Figure 12). These dates match the optical observations of PAR and NDVI well. The period of snow melting (1–2) is characterized by a very rapid increase in both *fAPAR_g_* and NDVI. During this period GPP increases quite slowly. The period after the main snow melt, when the buds of the birch trees are developing (3–4), is identical with the NDVI “shoulder” period. During this period GPP increases slowly, probably due to a low rate of understorey photosynthesis. *fAPAR_g_* has reached its maximum, and *fAPAR_c_* is relatively constant. In mid-June (5) the birch leaves are fully developed, and both GPP, NDVI and *fAPAR_c_* increase rapidly. They reach a maximum at DOY 183 (NDVI four days earlier than GPP), and level out during the middle of the peak season. In the autumn, GPP starts decreasing ca. 18 days earlier than NDVI and *fAPAR_c_*. Leveling out of GPP matches the date of yellowing of leaves (6), and NDVI reaches its minimum value. After this date, NDVI increases again, concurrently with *fAPAR_g_*, and probably as a consequence of exposure of the green understorey due to shedding of the birch leaves. GPP remains low but positive throughout this period, indicating some understorey vegetation productivity. When the temperature falls below zero degrees NDVI drops rapidly to below 0.2. During the winter season the optical data sets are very noisy. During the growing season, however, it can be noted that the NDVI curve is less noisy than any of the other curves, including GPP. The green fields in Figure 12 denote the vegetation period according to the optical measurements: (A) the period dominated by understorey growth; (B) the period from bud-burst to full leaf development; (C) the period of leaves developing into a full canopy; (D) the period of mature canopy; (E) the period of discoloration and start of defoliation of the canopy; and (F) the period of re-exposure of ground vegetation due to defoliation.

## 6. Discussion

### 6.1. Validity and Use of the Data

The measurements, and careful data processing, enabled us to portray the vegetation phenology of the studied sites accurately. Though the optical flux measurements contained some noise, the derived NDVI curves were generally smooth during the growing season. The good correspondence between growing-season NDVI from our towers with data from MODIS was encouraging. The network has radiation sensors and data loggers with very low power consumption that can run on backup batteries during power failures. The measurements therefore provide simple and robust estimates of growing season phenology that are useful additions to flux measurements and will greatly improve our understanding of ecosystem processes. The relatively low cost of optical measurements means that they can be extended to cover more ecosystems.

Our computations demonstrated that the spectral reflectance measured with large FOV sensor is close to that of directional measurements with infinitesimal FOV. This is advantageous since a larger footprint area is monitored, and a stronger canopy signal is obtained compared to narrow-FOV measurements. The large proportion of canopy in the FOV means that phenophase transition points and subtle changes during the growing season are easily observed. The measurements are affected by anisotropy, and the radiative-transfer model simulations showed that our NDVI values are likely to be higher than those measured at nadir. However, by pointing the sensor in east-west direction and by selecting observations close to mid-day we avoid variations due to the regions of maximum and minimum reflectance along the sun principal plane (hotspot and dark-spot, respectively). The large-FOV sampling averages out anisotropy variations within the footprint. Further simulations are necessary to investigate this for a larger number of canopy configurations and a variety of measurement situations, e.g., varied sun zenith angles and relative azimuth angles. We were not able to measure with the same sensor FOV at Norunda forest as at the other sites, and this is likely to introduce some problem when directly comparing data across the sites. However, the error is small as we avoid looking in the direction of the principal plane.

We noted large dark-current drift in our narrow-band measurements, particularly during wintertime due to weak incoming light. Temperature drift is a serious noise source for narrow-band spectral measurement in locations with large temperature variability, such as high latitude or altitude regions. Under these conditions, laboratory-calibrated sensor offsets may not be useful, and great care is necessary to correct for the drift. Although our use of night-time observations for drift compensation improved the time series, we have not been able to test if the temperature-drift relationship holds for daytime conditions.

The measurements have revealed some interesting phenological features, for example, the shoulder period of the NDVI after the snow melting period, when ground vegetation recovers from winter. This is the period when water from snow melt becomes available to the vegetation. The brief persistence of such surface water may produce lower NDVI values, possibly canceling out the NDVI increase from vegetation growth. This may explain the slight decrease in NDVI noted at some sites during this period. Another interesting feature was the very rapid increase in NDVI at the Stordalen mire during the snow-melting period, when the values increased from 0.04 to 0.44 within only 2 days, mainly due to the fact that the reflectance of red light decreased much faster than that of NIR. The simultaneous decrease in PAR reflectance was relatively slower. Apart from the fact that the two sensors are spectrally different, the discrepancy could be explained by the fact that the 60° FOV sensor and the hemispherical sensor were looking at different targets. The former was looking at a defined target area, whereas the latter was looking at an unlimited area. The onset of the PAR reflectance decrease was earlier than that of the NDVI, possibly caused by different snow melt rates near and farther away from the sensors.

The partitioning of PAR fluxes demonstrated how ground vegetation at the Abisko forest was able to take advantage of the brief period between the snow melt (ending on DOY 130) and when the leaves of the overstorey were fully developed (DOY 183). During this period of low shade, the environment was suitable for growth, and the ground vegetation had an opportunity to absorb more PAR than the tree canopy, as evidenced by the higher *fAPAR_g_* than *fAPAR_c_* in Figure 10 (Left). Some herbs and grasses in Swedish forests can grasp this opportunity to develop from sprouting to flowering and seeding in such a short time period.

A likely source of noise in our optical sampling network is the effect of raindrops, snow, and dust on the upward-looking sensors, which might lead to a negative bias in the irradiance measurement. Though we could not confirm it, this would overestimate reflectance, and since this influence would be greater for the NIR band than for the red band, would introduce some positive bias in the tower NDVI measurements. During the winter our ground sensors were indeed covered by snow during some periods.

### 6.2. Considerations for Expanding the Measurements in Space and Time

There is a need to expand the measurements to additional sites, and to cover longer time intervals. For long-term monitoring it is necessary to ensure proper calibration at regular intervals to avoid drifts or degradation due to exposure of rain, snow, frost, wind and dust. We strongly recommend users to carry out compensation for dark-current drift. This can be problematic at high-latitude locations where there may be light even during the middle of the night. The dark drift effect on daytime measurements should be further analyzed. Another possible sensor drift is magnitude drift. Reflectance can be cross-calibrated by using a field spectroradiometer, as shown by Ryu [51]. However, calibrating the absolute values of irradiance and radiance in the field is not possible, and to ensure correct absolute values of these quantities sensors will have to be recalibrated in a laboratory at regular intervals. Another possible source of error is spectral drift. There is no easy way to calibrate this in the field, and calibration in a laboratory is needed. Our sensor manufacturer recommends calibration of their light sensors every two years.

We suggest mounting additional downward and upward PAR sensors in the forest stands to enable computation of all four PAR fluxes at all sites, hence enable accurate determination of canopy PAR absorption. These data will aid in the development of light-use efficiency models, and for further understanding of satellite-data driven productivity models. For the deciduous stands, both NDVI and absorbed PAR were strongly related to phenology, though the NDVI signal was less noisy during the growing season. For the coniferous stands, transmitted PAR was not sensitive to seasonal canopy variations, whereas reflected PAR showed some seasonal variation. Based on only one year of data, NDVI appeared somewhat better for observing coniferous phenology than any of the other radiation quantities.

## 7. Conclusions

We have set up a tower-based network for spectral measurements that has generated a first year of measurements in narrow bands of red, NIR, and two green bands, and broadband measurements of PAR. The measurements were used to derive reflectances in corresponding bands, NDVI, and fractions of PAR. The network currently is co-located with eddy covariance towers, and covers two coniferous forests, one deciduous forest and two peatland sites. We plan to extend this spectral network to cover more ecosystems in Fennoscandia. The measurements add to the knowledge of ecosystem processes for more accurate determination of phenological transitions and primary productivity. The data also provide information for validating satellite products and calibrating new algorithms for such products. The results in this paper show that there is great potential for characterizing subtle variations in land surface phenology at high temporal resolution using a combination of PAR and multi-spectral measurements. Our computations showed that NDVI sensors mounted in off-nadir directions away from the principal plane were only moderately affected by variations in anisotropic reflectance.

We decomposed the ecosystem-reflected PAR fluxes into contributions from forest canopy and ground (understorey layers, including vegetation, litter fall, bare soil, and snow), and partitioned the total absorbed PAR into canopy-absorbed and ground-absorbed. This enabled detailed analysis of PAR reflectance changes and PAR absorption of the different ecosystem components throughout the year, thus providing data for better calibration of GPP models. We showed that the “true” canopy transmittance and interceptance of PAR are different from those computed from direct measurement. This discrepancy can be large for bright backgrounds.

The tower-based NDVI measurements agreed very well with MODIS data during the growing season, and were much smoother. The frequency of good tower measurements is much higher than that of MODIS, allowing for a more detailed analysis of phenological events. The data will thus enable thorough evaluation of the methods used in current remote sensing practice for satellite data, e.g., methods for temporal compositing and data smoothing.

## Figures and Tables

**Table 1.** Sample site information (location in decimal degrees).

**Variable**	**Abisko forest**	**Stordalen mire**	**Norunda forest**	**Fäjemyr bog**	**Hyytiälä forest**
Latitude	68.36	68.36	60.09	56.27	61.85
Longitude	18.80	19.05	17.48	13.55	24.29
Elevation a.s.l. (m)	340	360	70	140	170
Climate zone	Sub-arctic	Sub-arctic	Boreal	Temperate	Boreal
Mean annual temperature (°C)	−0.7	−0.7	5.5	6.2	3.5
Annual Precipitation (mm)	300	300	527	700	640
Ecosystem	Deciduous forest	Sub-arctic mire	Coniferous forest	Peat bog	Coniferous forest
Dominant Vegetation	Sub-arctic birch forest (Betula pubescens)	Mire with discontinuous permafrost palsas; Eriophorum spp.	Mixed coniferous forest (Pinus sylvestris and Picea abies)	Ombotrophic peat bog with scatterd dwarf pine (Pinus sylvestris)	Pine forest (Pinus sylvestris)
Tree density (stem/ha)	1,300	N/A	600	N/A	2,500
Tree height (m)	4	N/A	25	N/A	15
Sensor types^a^	SKR-1850A, JYP-1000	SKR-1850A, JYP-1000	SKR-1800, JYP-1000	SKR-1800, JYP-1000	SKR-1800, JYP-1000

a The JYP-1000 sensor from SDEC France (http://www.sdec-france.com/) is used for PAR measurement, see further details in Section 2.2.

**Table 2.** Specification of the sensor characteristics.

**Manufacturer**	**Type**	**Central wavelength (nm)**	**Band width (nm)**	**Accuracy (nm)**	**FOV of directional sensor**
SKYE	SKR-1850A	530, 570, 650, 870	10	±2 nm	60°
SKYE	SKR-1800	650, 860	50	±2 nm	60°; 25° (Norunda forest)
SDEC	JYP-1000	550	260	N/A	180°

**Table 3.** Geometry information multispectral measurements or reflected radiation.

**No.**	**Name**	**Height above canopy (m)**	**View azimuth angle (degrees)**	**Sensor FOV**	**Approximate footprint size at canopy level (m^2^)**
1	Abisko forest	8	261	60	1,961
2	Abisko mire	8	313	60	1,961
3	Norunda forest	38	135	25	1,384
4	Fäjemyr bog	10	276	60	3,064
5	Hyytiälä forest	15	70	60	6,894

**Table 4.** Currently installed sensors for estimation of PAR fluxes. Abbreviations of PAR fluxes are given in the text.

**No.**	**Name**	**No. of PAR sensors**			
		*PAR*_0_	*RPAR_eco_*	*TPAR*	*RPAR_g_*
1	Abisko forest	1	1	4	1
2	Stordalen mire	1	1	-	-
3	Norunda forest	1	1	14	-
4	Fäjemyr bog	1	1	-	-
5	Hyytiälä forest	-	-	-	-

**Table 5.** Main parameters of the Abisko forest stand for the FRT model.

**Parameter**	**Unit**	**Value**
Tree density	Trees/m^2^	0.13
Tree height	m	6.95
Crown length	m	6.95
Crown radius	m	3.28
Trunk diameter at breast height	cm	12
Dry weight of leaves	kg/tree	0.583

**Table 6.** Formulae for fractions of PAR absorption and interception, as well as for PAR reflectance and transmittance. *τPAR_m_* is the measured canopy transmittance, *RPAR_c_* is the purely canopy-reflected PAR which is a part of the total reflected PAR, *RPAR_eco_*, after subtracting fluxes that directly reach the sensor from ground reflection, or after canopy transmission. *RPAR_g_* is the ground reflected PAR, including reflection from bare soil and ground vegetation below the tree canopy. See Section 2.2 for explanation of measured PAR quantities.

**Target**	**Reflectance (or transmittance)**	**Fractions of absorption (or interception)**	**Reference**
Canopy^a^	$\tau {\mathrm{PAR}}_{m}=\frac{\mathrm{TPAR}}{{\mathrm{PAR}}_{0}}$	$\mathrm{fIPA}R_{m}=\frac{{\mathrm{PAR}}_{0}-\mathrm{TPAR}}{{\mathrm{PAR}}_{0}}=1-\tau {\mathrm{PAR}}_{m}$	[45]
Canopy	$\mathrm{rPA}R_{c}=\frac{\mathrm{RPA}R_{c}}{{\mathrm{PAR}}_{0}}$	$\begin{array}{c} \mathrm{fAPA}R_{c}=\frac{{\mathrm{PAR}}_{0}+\mathrm{RPA}R_{g}-\mathrm{TPAR}-\mathrm{RPA}R_{\mathrm{eco}}}{{\mathrm{PAR}}_{0}}\\ =1-\mathrm{rPA}R_{\mathrm{eco}}-(1-\mathrm{rPA}R_{g})\cdot \tau {\mathrm{PAR}}_{m} \end{array}$	[45]
Ground	$\mathrm{rPA}R_{g}=\frac{\mathrm{RPA}R_{g}}{\mathrm{TPAR}}$	$\begin{array}{c} {\mathrm{fAPAR}}_{g}=\frac{\mathrm{TPAR}-{\mathrm{RPAR}}_{g}}{{\mathrm{PAR}}_{0}}\\ \;\;\;\;\;\;\;\;\;\;\;\;\;\;\;\;\;\;\;\;\;\;\;\;\;\;\;\;\;\;\;\;\;\;\;\;\;\;\;\;\;\;\;\;=(1-\mathrm{rPA}R_{g})\cdot \tau {\mathrm{PAR}}_{m} \end{array}$	[46]
Eco-system	$\mathrm{rPA}R_{\mathrm{eco}}=\frac{\mathrm{RPA}R_{\mathrm{eco}}}{{\mathrm{PAR}}_{0}}$	$\begin{array}{c} \mathrm{fAPA}R_{e}=\frac{{\mathrm{PAR}}_{0}-\mathrm{RPA}R_{\mathrm{eco}}}{{\mathrm{PAR}}_{0}}\\ \;\;\;\;\;\;\;\;\;\;\;\;\;\;\;\;\;\;\;\;\;\;\;\;\;\;\;\;\;\;\;\;\;\;\;\;\;\;\;\;\;\;\;\;\;=\mathrm{fAPA}R_{c}+\mathrm{fAPA}R_{g}\\ \;\;\;\;\;\;\;\;\;\;\;\;=1-\mathrm{rPA}R_{\mathrm{eco}} \end{array}$	[47]

a The measured canopy transmission and inception have contributions from the ground. Quantities that have been corrected for this are shown in the text.

**Figure A1.**
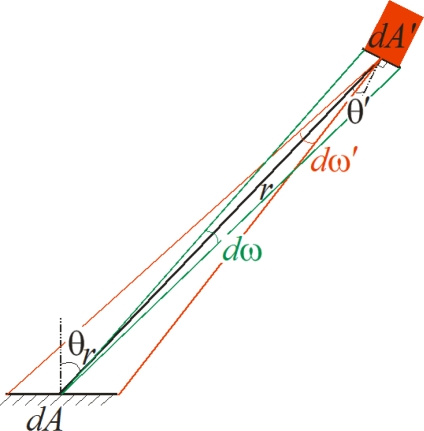
Schematic diagram of a light beam from the ground small area *dA* to the sensor aperture area *dA′*.

## Appendix 1. Deduction of the Mean Footprint Reflectance (HCRF)

The footprint reflectance is computed from measured downward irradiance and upward radiance. Measuring the hemispherical irradiance *E* [μmol·s^−1^·m^−2^] is quite straightforward by using a 180°-FOV upward-looking sensor. A cosine correction diffuser is used to ensure that the quantum response of the sensor to the photon spectrum is the same in all hemispherical directions across the sensor aperture. Derivation of the upwelling reflected radiance measured by an obliquely downward-looking sensor is less straight-forward. We will discuss this in detail in the following section.

### Sensor Response

A light flux (power) radiated from a small area *dA* to another small area *dA′* is given by Lambert’s Cosine Law [55]:

(A1) $$d\Phi_{r}=\frac{L_{\theta }\cdot dA\cdot \text{cos}\left(\theta_{r}\right)\cdot dA^{\prime }\cdot \text{cos}\left(\theta^{\prime }\right)}{r^{2}},$$

where *L_θ_*[μmol·s^−1^·m^−2^·sr^−1^] is the radiance from *dA* with an angle *θ_r_* to the normal of *dA*, falling onto the area *d*A′ with an angle *θ′* to the normal of *d*A′, and *r* is the distance between the two small areas (Figure A1). The projected solid angle subtended by *d*A is denoted by *d*ω′ and the solid angle subtended by *d*A′ is denoted by *d*ω, *i.e.*, *d*ω = (*d*A′ · cos (*θ′*))/*r*^2^, and *d*ω′a(*d*A·cos(*θ_r_*))/*r*^2^, then:

(A2) $$d\Phi_{r}=L_{\theta }\cdot dA\cdot \text{cos}\left(\theta_{r}\right)d\omega =L_{\theta }\cdot dA^{\prime }\cdot \text{cos}\left(\theta^{\prime }\right)\cdot d\omega^{\prime }.$$

The measurement given by a light sensor, *i.e.*, the sensor response, is the total integrated flux of *dΦ_r_* over the footprint area *A* and the sensor aperture *A′* [52]. From the aspect of sensor, the sensor response is:

(A3) $$\Phi_{r}=\int_{A^{\prime }}\int_{A}d\Phi_{r}=\int_{A^{\prime }}\int_{\Omega_{A}}L_{\theta }\cdot dA^{\prime }\cdot \text{cos}\left(\theta^{\prime }\right)d\omega^{\prime }.$$

Note that the integration over the footprint area *A* is the same as over the solid angle Ω*_A_* subtended by the footprint *A*, or in other words, subtended by the sensor FOV, depending on what variable is used in the integrand. We also assume that the sensor is a simple aperture, with equal response to radiation at any point across its surface and from any direction [53], *i.e.*, a cosine-corrected sensor. The mean radiance of the footprint measured by such a sensor is given by the definition of radiance:

(A4) $${\bar{L}}_{r}=\frac{\Phi_{r}}{\int_{A^{\prime }}\int_{\Omega_{A}}dA^{\prime }\cdot \text{cos}\left(\theta^{\prime }\right)\cdot d\omega^{\prime }}=\frac{\int_{A^{\prime }}\int_{\Omega_{A}}L_{\theta }\cdot dA^{\prime }\cdot \text{cos}\left(\theta^{\prime }\right)\cdot d\omega^{\prime }}{\int_{A^{\prime }}\int_{\Omega_{A}}dA^{\prime }\cdot \text{cos}\left(\theta^{\prime }\right)\cdot d\omega^{\prime }}.$$

Since *d*A′ and *d*ω′ are independent of each other, and the projected area cos(*θ′*) · *d*A′ is independent of the other variables in the equation, we take out the projected area and get:

(A5) $${\bar{L}}_{r}=\frac{\int_{\Omega_{A}}L_{\theta }\cdot d\omega^{\prime }}{\int_{\Omega_{A}}d\omega^{\prime }}.$$

The projected solid angle is denoted by Ω, and:

(A6) $$\Omega =\int_{\Omega_{A}}d\omega^{\prime }=\int_{A}\frac{\text{cos}\left(\theta_{r}\right)}{r^{2}}\cdot dA,$$

where *d*A can be further expressed as *d*A = *dxdy* in a Cartesian coordinate system and (*x*, *y*) is a point within the footprint ellipse.

### Footprint Reflectance

Let *f*(*θ_r_*) [sr^−1^] be the hemispherical-directional reflectance distribution function (HDRDF) of the small area *d*A to the direction *θ_r_*. Note: for clarity purpose, we do not express azimuth angle or wavelength explicitly in the denotation. The reflected radiance of a hemispherical incoming radiance *E* onto *d*A is:

(A7) $$L_{\theta }=E\cdot f\left(\theta_{r}\right).$$

Rewrite Equation (A5) by using Equation (A7):

(A8) $${\bar{L}}_{r}=\frac{\int_{\Omega_{A}}E\cdot f\left(\theta_{r}\right)\cdot d\omega^{\prime }}{\Omega }.$$

The footprint hemispherical-conical reflectance factor (HCRF) is computed by:

$$\bar{\rho }=\frac{\pi {\bar{L}}_{r}}{E}=\frac{\pi \int_{\Omega_{A}}E\cdot f\left(\theta_{r}\right)\cdot d\omega^{\prime }}{E\cdot \Omega }=\frac{\int_{\Omega_{A}}\pi f\left(\theta_{r}\right)\cdot d\omega^{\prime }}{\int_{\Omega_{A}}d\omega^{\prime }}.$$

Writing this in the Cartesian coordinate system yields:

(A9) $$\bar{\rho }=\frac{\int_{A}\pi f\left(\theta_{r}\right)\cdot \frac{\text{cos}\left(\theta_{r}\right)}{r^{2}}\mathrm{dxdy}}{\int_{A}\frac{\text{cos}\left(\theta_{r}\right)}{r^{2}}\mathrm{dxdy}}.$$

## Appendix 2. Computation of HCRF by Monte-Carlo Integration

In a Cartesian coordinate system with the origin at the footprint ellipse center, we write the ellipse as $\frac{x^{2}}{a^{2}}+\frac{y^{2}}{b^{2}}=1$, where *a* and *b* are semi-major and semi-minor axes respectively. It can be proved that *a* and *b* are functions of three variables of the observation geometry: sensor height *H*, sensor field-of-view angle *β*, and off-nadir angle *α*:

(A10) $$\begin{array}{c} a=H\cdot \frac{\text{tan}\left(\alpha +\frac{\beta }{2}\right)-\text{tan}\left(\alpha -\frac{\beta }{2}\right)}{2},\\ b=H\cdot \sqrt{{\left\{\frac{\text{cos}\left(\alpha \right)+\text{sin}\left(\alpha \right)\cdot l/H}{\text{cos}\left(\frac{\beta }{2}\right)}\right\}}^{2}-{\left(\frac{l}{H}\right)}^{2}-1,} \end{array}$$

where $l=H[\text{tan}\left(\alpha +\frac{\beta }{2}\right)+\text{tan}\left(\alpha -\frac{\beta }{2}\right)]/2$. The integral in the HCRF formula in Equation (A9) can be computed by Monte-Carlo integration as:

(A11) $$\bar{\rho }=E\left[\frac{\pi f\left(\theta_{r}\right)\cdot \text{cos}\left(\theta_{r}\right)\cdot 𝟙\left(\frac{x^{2}}{a^{2}}+\frac{y^{2}}{b^{2}}\leq 1\right)}{f\left(x\right)f\left(y\right)\cdot r^{2}}\right]/E\left[\frac{\text{cos}\left(\theta_{r}\right)\cdot 𝟙\left(\frac{x^{2}}{a^{2}}+\frac{y^{2}}{b^{2}}\leq 1\right)}{f\left(x\right)f\left(y\right)\cdot r^{2}}\right],$$

where the indicator function 𝟙 if the random point (*x*, *y*) falls in the ellipse (including border), and 0 otherwise. *f*(*x*) and *f*(*y*) are probability density functions of *x* and *y* respectively. *E*(·) is the expectation of the integrand, which is approximated by the mean from N random samples, if N is large enough.

It can be proved that:

(A12) $$\begin{array}{c} f(x)=\frac{1/H}{\beta }\cdot \frac{1}{1+\frac{{\left(x-l\right)}^{2}}{H^{2}}},\\ f(y)=\frac{0.5/\sqrt{l^{2}+H^{2}}}{\text{arctan}\;(\frac{b}{\sqrt{l^{2}+H^{2}}})}\cdot \frac{1}{1+\frac{y^{2}}{l^{2}+H^{2}}}\cdot \end{array}$$

Therefore the marginal distributions of *x* and *y* are Cauchy distributions. Their corresponding random variables can be generated by:

$$\begin{array}{c} x=l-H\cdot \text{tan}\left(\theta_{x}\right),\\ y=\sqrt{l^{2}+H^{2}}\cdot \text{tan}\left(\theta_{y}\right), \end{array}$$

where *θ_x_* and *θ_y_* are sampled from a uniform distribution:

$$\begin{array}{c} \theta_{x}\in U\left(\alpha -\frac{\mathrm{FOV}}{2},\alpha +\frac{\mathrm{FOV}}{2}\right),\;\text{and}\\ \theta_{y}\in U\left(-\text{arctan}\left(\frac{b}{\sqrt{l^{2}+H^{2}}}\right),\text{arctan}\left(\frac{b}{\sqrt{l^{2}+H^{2}}}\right)\right). \end{array}$$

## Appendix 3. Separating Pure Canopy PAR Reflection from the Total Reflected PAR

We assume that the canopy is macro-homogeneous and unlimited large, and canopy reflectance and canopy transmittance are all isotropic. Any kind of gap is allowed in our simplified canopy model, including gaps between tree crowns, within tree crowns, between leaves, or even between cellular gaps. We attribute the canopy transmission of PAR to the effect of all these gaps, without considering canopy scattering. The possible error due to this [54] is regarded as minor for snow-free conditions. An incoming PAR beam onto the canopy undergoes multiple transmission and reflection between canopy and ground (Figure A2).

The downward-looking sensor above the canopy measures:

$$\begin{array}{c} \mathrm{TPA}R_{m}=T_{0}+T_{1}+T_{2}+\cdots \\ ={\mathrm{PAR}}_{0}\cdot \tau \mathrm{PAR}+{\mathrm{PAR}}_{0}\cdot \tau \mathrm{PAR}\cdot (\mathrm{rPA}R_{g}\cdot \mathrm{rPA}R_{c})+{\mathrm{PAR}}_{0}\cdot \tau \mathrm{PAR}\cdot {\left(\mathrm{rPA}R_{g}\cdot \mathrm{rPA}R_{c}\right)}^{2}\cdots \\ \;\;\;\;\;\;\;\;\;\;\;\;={\mathrm{PAR}}_{0}\cdot \frac{\tau \mathrm{PAR}}{1-\mathrm{rPA}R_{c}\cdot \mathrm{rPA}R_{g}}, \end{array}$$

where *rPAR_c_* is pure canopy reflectance, *τPAR* is the “true” canopy transmittance, and *rPAR_g_* is the “true” ground reflectance.

Thus:

(A13) $$\mathrm{rPA}R_{\mathrm{eco}}=\mathrm{rPA}R_{c}+\frac{\tau \mathrm{PA}R^{2}\cdot \mathrm{rPA}R_{g}}{1-\mathrm{rPA}R_{c}\cdot \mathrm{rPA}R_{g}}.$$

The upward-looking sensor below canopy measures:

$$\begin{array}{c} \mathrm{TPA}R_{m}=T_{0}+T_{1}+T_{2}+\cdots \\ ={\mathrm{PAR}}_{0}\cdot \tau \mathrm{PAR}+{\mathrm{PAR}}_{0}\cdot \tau \mathrm{PAR}\cdot (\mathrm{rPA}R_{g}\cdot \mathrm{rPA}R_{c})+{\mathrm{PAR}}_{0}\cdot \tau \mathrm{PAR}\cdot {\left(\mathrm{rPA}R_{g}\cdot \mathrm{rPA}R_{c}\right)}^{2}\cdots \\ \;\;\;\;\;\;\;\;\;\;\;\;\;={\mathrm{PAR}}_{0}\cdot \frac{\tau \mathrm{PAR}}{1-\mathrm{rPA}R_{c}\cdot \mathrm{rPA}R_{g}}. \end{array}$$

Thus, the measured transmittance of PAR has the following relation with the “true” PAR transmittance:

(A14) $$\tau {\mathrm{PAR}}_{m}=\frac{\tau \mathrm{PAR}}{1-\mathrm{rPA}R_{c}\cdot \mathrm{rPA}R_{g}}.$$

The downward-looking sensor below canopy measures ground reflection:

$$\begin{array}{c} \mathrm{RPA}R_{g\_m}=Rg_{0}+Rg_{1}+Rg_{2}+\cdots \\ ={\mathrm{PAR}}_{0}\cdot \tau \mathrm{PAR}\cdot r{\mathrm{PAR}}_{g}+{\mathrm{PAR}}_{0}\cdot \tau \mathrm{PAR}\cdot ({\mathrm{rPAR}}_{g}\cdot {\mathrm{rPAR}}_{c})\cdot {\mathrm{rPAR}}_{g}+{\mathrm{PAR}}_{0}\cdot \tau \mathrm{PAR}\cdot {\left(\mathrm{rPA}R_{g}\cdot {\mathrm{rPAR}}_{c}\right)}^{2}\cdot {\mathrm{rPAR}}_{g}\cdots \\ ={\mathrm{PAR}}_{0}\cdot \frac{\tau \mathrm{PAR}\cdot \mathrm{rPA}R_{g}}{1-\mathrm{rPA}R_{c}\cdot \mathrm{rPA}R_{g}}. \end{array}$$

Thus, the measured ground reflectance of PAR has the following relation with the “true” ground reflectance of PAR:

(A15) $$\mathrm{rPA}R_{g\_m}=\frac{\mathrm{rPA}R_{g\_m}}{\mathrm{TPAR}}=\mathrm{rPA}R_{g},$$

and the measured ground reflectance is the same as the “true” reflectance. From Equations (A13–A15), we get the expressions of pure canopy reflectance *rPARc* and “true” canopy transmittance *τPAR*:

(A16) $$\mathrm{rPA}R_{c}=\frac{\mathrm{rPA}R_{\mathrm{eco}}-\tau \mathrm{PA}R_{m}^{2}\cdot \mathrm{rPA}R_{g}}{1-\tau \mathrm{PA}R_{m}^{2}\cdot \mathrm{rPA}R_{g}^{2}},$$

(A17) $$\tau \mathrm{PAR}=\tau {\mathrm{PAR}}_{m}\cdot (1-\mathrm{rPA}R_{c}\cdot \mathrm{rPA}R_{g}).$$

The canopy intercepted PAR is:

$$\begin{array}{c} \mathrm{IPAR}={\mathrm{PAR}}_{0}-T_{0}+(Rg_{0}-R_{1})+(Rg_{1}-R_{2})+\cdots \\ ={\mathrm{PAR}}_{0}-T_{0}+(Rg_{0}+Rg_{1}+\cdots )-(R_{1}+R_{2}+\cdots )\\ ={\mathrm{PAR}}_{0}(1-\tau \mathrm{PAR})+{\mathrm{PAR}}_{0}\cdot \frac{\tau \mathrm{PAR}\cdot \mathrm{rPA}R_{g}}{1-\mathrm{rPA}R_{c}\cdot \mathrm{rPA}R_{g}}-{\mathrm{PAR}}_{0}\cdot \frac{\tau \mathrm{PA}R^{2}\cdot \mathrm{rPA}R_{g}}{1-\mathrm{rPA}R_{c}\cdot \mathrm{rPA}R_{g}}. \end{array}$$

Finally, we get the *fIPAR*:

(A18) $$fI\mathrm{PAR}=(1-\tau \mathrm{PAR})\cdot (1+\tau {\mathrm{PAR}}_{m}\cdot \mathrm{rPA}R_{g}).$$
